# Glycated hemoglobin and dynapenia in community- dwelling older people with and without diabetes: A cross-sectional study

**DOI:** 10.1007/s40200-026-01868-w

**Published:** 2026-02-24

**Authors:** André Luiz da Silva, Daniela Braga Lima, Gustavo Andrade Brancaglion, Guilherme Eustáquio Furtado, Ligiana Pires Corona, Tábatta Renata Pereira de Brito

**Affiliations:** 1 Faculty of Nutrition, Federal University of Alfenas, Rua Gabriel Monteiro da Silva, 700, Centro, Alfenas, Minas Gerais CEP: 37130-000 Brazil; 2 Faculty of Pharmaceutical Sciences, Federal University of Alfenas, Rua Gabriel Monteiro da Silva, 700, Centro, Alfenas, Minas Gerais CEP: 37130-000 Brazil; 3 University Polytechnic of Coimbra, Rua da Misericórdia, Lagar dos Cortiços S. Martinho do Bispo, Coimbra, 3045-093 Portugal; 4 SPRINT - Center for Innovation and Research in Sport, Physical Activity & Health, Rua da Misericórdia, Lagar dos Cortiços S. Martinho do Bispo, Coimbra, 3045-093 Portugal; 5 Research Centre for Natural Resources Environment and Society (CERNAS), Polytechnic Institute of Coimbra, Bencanta, Coimbra, 3045-601 Portugal; 6 School of Applied Sciences, University of Campinas (UNICAMP), Limeira, SP Brazil

**Keywords:** Aged, Diabetes mellitus, Muscle strength, Glycated hemoglobin, Handgrip strength

## Abstract

**Purpose:**

Dynapenia, an age-related loss of muscle strength, is associated with adverse outcomes in older people. Elevated glycated hemoglobin (HbA1c) levels may accelerate muscle decline, especially in individuals with diabetes mellitus. This study aimed to determine whether HbA1c levels are associated with dynapenia in community-dwelling older people, and whether this association differs between those with and without diabetes mellitus.

**Methods:**

A population-based cross-sectional study was conducted with a probabilistic sample of 404 community-dwelling older people in southern Brazil. Data collection included home interviews, physical assessments, and laboratory tests. Dynapenia was defined as mean handgrip strength below 26 kg for men and 16 kg for women. HbA1c was measured by high-performance liquid chromatography. Multiple logistic regression was used to assess the association between HbA1c and dynapenia, stratified by diabetes status.

**Results:**

The sample was predominantly female (72.0%) and aged 60–69 years (44.3%). The prevalence of diabetes mellitus was 36.4%, and dynapenia was observed in 23.5% of participants. In multivariable logistic regression analysis, higher HbA1c levels were independently associated with dynapenia among older people with diabetes mellitus (adjusted odds ratio = 1.34; 95% confidence interval: 1.05–1.71), regardless of sex, age, body mass index, and physical activity.

**Conclusion:**

Glycemic control is crucial to prevent muscle strength decline in older people with diabetes mellitus. Monitoring muscle strength should be part of clinical care to reduce functional impairment and adverse outcomes in this population.

## Introduction

Glycated hemoglobin (HbA₁c) is widely used as a marker of glycemic control and is also recognized as a biomarker of aging [1, 2]. It is considered the gold standard marker of long-term glycemic control because it reflects average blood glucose levels over the previous two to three months, is less affected by short-term fluctuations, and has well-established associations with chronic complications of diabetes [3].

Skeletal muscle plays a key role in maintaining glucose homeostasis and is one of the primary sites for glucose uptake [4]. HbA₁c has been associated with reduced muscle strength in both diabetic and non-diabetic individuals [5, 6]. Moreover, chronic hyperglycemia can impair muscle integrity and innervation, contributing to musculoskeletal dysfunction [7].

Among the major risk factors for functional decline in older adults is dynapenia, defined as the age-related loss of muscle strength and power, independent of declines in muscle mass [8]. This condition compromises musculoskeletal function and is associated with an increased risk of disability, hospitalization, and mortality. In addition, low muscle strength is an independent predictor of adverse outcomes in older adults, underscoring the clinical relevance of dynapenia [9–11].

Studies indicate that persistently elevated HbA₁c levels increase the risk of dynapenia in both sexes [12, 13]. However, few investigations have assessed this effect in older people without a prior diabetes mellitus diagnosis, limiting our understanding of the preventive potential of glycemic control in community-dwelling populations.

It is estimated that by 2045, more than 783 million people will be living with diabetes mellitus worldwide, with a substantial proportion aged 60 years or older [14]. In Brazil, population aging has been accelerating rapidly, with older people accounting for 15.8% of the total population in 2022, representing a 46.6% increase compared to the 2010 census [15].

This demographic shift is associated with physiological changes during aging, such as reduced hormone production, increased oxidative stress, and impaired physical function [16], contributing to greater frailty among older individuals. Data from the Brazilian Longitudinal Study of Aging (ELSI-Brazil) indicate that 67.8% of older people present multimorbidity, reflecting an increasingly complex clinical profile [17].

Given this background, the present study aimed to investigate the association between HbA₁c levels and the occurrence of dynapenia in community-dwelling older people with and without a diagnosis of diabetes mellitus.

## Materials and methods

### Study design and setting

This is a population-based cross-sectional study with a quantitative and analytical approach, conducted in Alfenas, a municipality in the southern region of Minas Gerais, Brazil. The study design followed the Strengthening the Reporting of Observational Studies in Epidemiology (STROBE) guidelines [18], as part of a project funded by the Brazilian National Council for Scientific and Technological Development (CNPq) and the Research Support Foundation of the State of Minas Gerais (FAPEMIG), entitled “Association between low social support and telomere length in older people.”

### Participants

The study population consisted of individuals aged 60 years or older residing in the urban area of the municipality of Alfenas, Brazil, in 2019. Sample size calculation was performed for the larger project, considering an estimated prevalence of 50% for multiple outcomes, a 95% confidence level, a design effect of 1.17, and a total population of 10,797 older people, resulting in a required sample of 435 participants. To compensate for potential losses and the exclusion of participants without complete data for all variables included in the analyses, an additional 84 participants were included, totaling 519 interviewed individuals. After exclusions due to incomplete data, inconclusive laboratory results, or unavailability for blood collection, the final analytical sample comprised 404 participants, of whom 257 did not have a diagnosis of diabetes mellitus (63.6%) and 147 had diabetes mellitus (36.4%).

Inclusion criteria were: being 60 years of age or older and having sufficient cognitive ability to answer the questionnaire, as assessed by the interviewer. Individuals with permanent or temporary physical disabilities that prevented ambulation, except for those using walking aids, were excluded.

### Procedures and data collection

Data collection was conducted between July and December 2019, in two stages: a home interview including physical assessment, and a blood sample collection. Blood collection was performed at the Central Laboratory of Clinical Analyses (LACEN) of the Federal University of Alfenas (UNIFAL-MG) or at the participant’s home, according to availability.

Interviews were conducted by trained undergraduate and graduate students under faculty supervision. All participants were instructed to fast for at least 8 h prior to blood collection, which was performed by licensed professionals within a maximum of seven days after the interview.

### Study variables

#### Main independent variable

The main independent variable was glycated hemoglobin (HbA1c), measured by high-performance liquid chromatography (HPLC) using whole blood samples collected in EDTA tubes. Diabetes mellitus diagnosis was defined by self-report of a medical diagnosis and/or use of glucose-lowering medication.

#### Dependent variable

The variable outcome was dynapenia, assessed by handgrip strength (HGS) using a portable, calibrated SAEHAN SH5001 hydraulic dynamometer. The assessment was conducted at the participant’s home, with the individual seated, shoulder adducted and in a neutral position, elbow flexed at 90°, forearm in a neutral position, and wrist between 0 and 30° of extension. Three measurements were performed with the dominant arm, with at least one minute between attempts, and the mean value of the three trials was used for analysis. The cutoff points adopted to define dynapenia were < 26 kg for men and < 16 kg for women, these cutoff points and the handgrip strength assessment protocol are widely used in epidemiological studies and are supported by the Foundation for the National Institutes of Health (FNIH) Sarcopenia Project [19].

### Covariates

Covariates included sociodemographic, clinical, and behavioral variables: sex, age group, educational level, household income, living arrangements, cognitive decline (CASI-S) [20, 21], depressive symptoms (GDS-15) [22], multimorbidity [23], BMI (according to the Brazilian Ministry of Health classification) [24], smoking, alcohol consumption, physical activity, use of glucose-lowering medication, and dietary intake.

Dietary intake was assessed by frequency of consumption of food groups. The CASI-S was used to screen for cognitive decline (cutoff score < 23), and the GDS-15 was used to screen for depressive symptoms (cutoff score ≥ 6).

### Ethical aspects

This study was approved by the Research Ethics Committee (Approval No. 2.668.936, CAAE: 88953918.7.0000.5142). Participation in the study was contingent upon the older people providing informed consent, which was documented through the signing of a consent form. All procedures were carried out in strict adherence to the ethical principles outlined in the Declaration of Helsinki [25], as well as the guidelines set forth in Resolution 466/12 of the National Health Council for research involving human subjects [26].

### Statistical analysis

Data were double-entered into Microsoft Excel (version 2019) to minimize typing errors and subsequently analyzed using Stata software (version 17.0). The normality of continuous variables was assessed using the Kolmogorov–Smirnov test. As the data did not present a normal distribution, continuous variables were summarized using medians and 25th and 75th percentiles, while categorical variables were expressed as proportions. Group differences were assessed using Pearson’s chi-square test or Fisher’s exact test, as appropriate. Associations between HbA1c and dynapenia were examined using multiple logistic regression models, with crude and adjusted odds ratios (OR) and 95% confidence intervals (CI) estimated. A significance level of 5% was adopted for all analyses.

The univariable analysis aimed to identify all variables potentially associated with dynapenia. Subsequently, multivariable analysis was performed to assess the simultaneous effect of multiple variables in the model, allowing for control of confounding. During this adjustment process, it is expected that variables initially significant in the univariable analysis may lose statistical significance when considered jointly. Therefore, the criterion adopted was to retain in the final multivariable models only those variables that remained statistically significant after adjustment. Variables that lost significance when analyzed simultaneously were excluded from the final models. Model adequacy was assessed using receiver operating characteristic (ROC) curves, which demonstrated good discriminatory performance for both older people with diabetes mellitus and those without diabetes mellitus.

## Results

The sample of this study consisted of 404 older people residing in a municipality in the southern region of Minas Gerais, Brazil. Of these, 257 (63.6%) did not report a diagnosis of diabetes mellitus, whereas 147 (36.4%) had the condition. Dynapenia was present in 95 participants (23.5%), observed in 61 individuals without diabetes mellitus (15.1%) and in 34 individuals with diabetes mellitus (8.4%).

Glycated hemoglobin (HbA1c) levels showed a statistically significant difference only among participants with diabetes mellitus, with higher levels observed in those with dynapenia (*p* = 0.043). Among participants with diabetes mellitus, the median HbA1c was 7.4% (IQR: 6.5–9.0) in those with dynapenia, compared with 6.9% (IQR: 6.1–8.0) in those without dynapenia. (Table 1).

**Table 1:** Median HbA1c levels (with interquartile ranges) according to dynapenia status in older people without and with diabetes

Diabetes status	Dynapenia	Median	P25	P75	*p*-value*
**No diabetes**	No	5.7	5.4	5.9	0.423
	Yes	5.7	5.4	5.9	
**With diabetes**	No	6.9	6.1	8.0	0.043
	Yes	7.4	6.5	9.0	

P25: 25th percentile; P75: 75th percentile. *p-value calculated using the Mann-Whitney U test

Regarding the characterization of participants with and without diabetes mellitus in relation to the presence of dynapenia, Table 2 shows that among individuals without diabetes mellitus, dynapenia was more prevalent in men (*p* = 0.028), older participants (*p* < 0.001), those with lower educational attainment (*p* = 0.012), underweight individuals (*p* = 0.001), those with depressive symptoms (*p* = 0.001), cognitive decline (*p* < 0.001), and those who did not engage in physical activity (*p* = 0.001). An association was also found with lower consumption of meat and fruits.

**Table 2:** Socioeconomic, health, and lifestyle characteristics of older people with and without diabetes (*n* = 257), by dynapenia status

Variable	Without Diabetes				With Diabetes			
	Total n(%)	Dynapenia		p*	Total n(%)	Dynapenia		p*
		No n(%)	Yes n(%)			No n(%)	*Yes* n(%)	
Sex								
Male	69 (26.8)	46 (66.7)	23 (33.3)	0.028	44 (30.0)	28 (63.7)	16 (36.3)	0.013
Female	188 (73.2)	150 (79.8)	38 (20.2)		103 (70.0)	85 (82.5)	18 (17.5)	
Age group								
60–69 years	115 (44.8)	99 (86.1)	16 (13.9)	< 0.001	64 (43.5)	56 (87.5)	8 (12.5)	0.009
70–79 years	96 (37.3)	74 (77.1)	22 (22.9)		65 (44.2)	47 (72.3)	18 (27.7)	
≥ 80 years	46 (17.9)	23 (50.0)	23 (50.0)		18 (12.3)	10 (56.0)	8 (44.0)	
Years of schooling								
> 4 years	85 (35.6)	72 (84.7)	13 (15.3)	0.012	47 (33.6)	37 (78.8)	10 (21.2)	0.649
≤ 4 years	154 (64.4)	108 (70.1)	46 (29.9)		93 (66.4)	70 (75.0)	23 (25.0)	
Living arrangement								
Not living alone	199 (78.4)	151 (75.9)	48 (24.1)	0.941	122 (84.0)	91 (74.6)	31 (25.6)	0.079
Living alone	55 (21.6)	42 (76.4)	13 (23.6)		23 (16.0)	21 (91.0)	2 (9.0)	
Family income								
> 2 minimum wages	85 (35.3)	71 (83.5)	14 (16.5)	0.113	45 (32.4)	33 (73.4)	12 (26.4)	0.833
> 1 and ≤ 2 min. wages	100 (41.5)	73 (73.0)	27 (27.0)		69 (49.6)	54 (78.3)	15 (21.7)	
≤ 1 minimum wage	56 (23.2)	39 (69.6)	17 (30.4)		25 (18.0)	19 (76.0)	6 (24.0)	
Multimorbidity								
No	104 (41.4)	79 (76.0)	25 (24.0)	0.867	12 (8.3)	5 (42.0)	7 (58.0)	0.003
Yes	147 (58.6)	113 (76.9)	34 (23.1)		133 (91.7)	106 (80.0)	27 (20.0)	
Depressive symptoms								
No symptoms	170 (66.1)	140 (82.4)	30 (17.6)	0.001	94 (64.0)	78 (83.0)	16 (17.0)	0.019
With symptoms	87 (33.9)	56 (64.4)	31 (35.6)		53 (36.0)	35 (66.0)	18 (34.0)	
Cognitive decline								
No	183 (71.2)	152 (83.0)	31 (17.0)	< 0.001	103 (70.5)	86 (83.5)	17 (16.5)	0.003
Yes	74 (28.8)	44 (59.5)	30 (40.5)		43 (29.5)	26 (60.5)	17 (39.5)	
BMI (kg/m²)								
Underweight	34 (13.4)	18 (53.0)	16 (47.0)	0.001	11 (7.6)	9 (82.0)	2 (18.0)	0.492
Normal	83 (32.7)	68 (82.0)	15 (18.0)		45 (31.0)	32 (71.1)	13 (28.9)	
Overweight	137 (53.9)	110 (80.2)	27 (19.8)		89 (61.9)	71 (79.8)	18 (20.2)	
Physical activity								
No	168 (68.3)	119 (70.9)	49 (29.1)	0.001	96 (69.0)	67 (69.8)	29 (30.2)	0.003
Yes	78 (31.7)	70 (89.8)	8 (10.2)		43 (31.0)	40 (93.0)	3 (7.0)	
Smoking								
No	222 (87.1)	169 (76.1)	53 (23.9)	0.963	133 (91.0)	103 (77.5)	30 (22.5)	0.504
Yes	33 (12.9)	25 (75.8)	8 (24.2)		13 (9.0)	9 (69.3)	4 (30.7)	
Alcohol consumption								
No	167 (67.1)	117 (70.1)	50 (29.9)	0.001	111 (77.0)	81 (73.0)	30 (27.0)	0.077
Yes	82 (32.9)	73 (89.0)	9 (11.0)		33 (23.0)	29 (88.0)	4 (12.0)	
Milk consumption								
Yes	182 (70.8)	137 (75.3)	45 (24.7)	0.561	106 (72.6)	81 (76.4)	25 (23.6)	0.890
No	75 (29.2)	59 (78.7)	16 (21.3)		40 (27.4)	31 (77.5)	9 (22.5)	
Meat consumption								
Yes	192 (74.7)	157 (81.8)	35 (18.2)	< 0.001	109 (74.6)	83 (76.2)	26 (23.8)	0.781
No	65 (25.3)	39 (60.0)	26 (40.0)		37 (25.4)	29 (78.4)	8 (21.6)	
Fruit consumption								
Yes	192 (75.3)	155 (81.0)	37 (19.0)	0.002	118 (81.0)	91 (77.1)	27 (22.9)	0.812
No	63 (24.7)	39 (62.0)	24 (38.0)		28 (19.0)	21 (75.0)	7 (25.0)	
Legume consumption								
Yes	232 (91.0)	177 (76.3)	55 (23.7)	0.799	137 (94.5)	105 (76.7)	32 (23.3)	0.915
No	23 (9.0)	17 (74.0)	6 (26.0)		8(5,5)	6(75)	2(25)	
Diabetes medication use								
Yes	—	—	—	—	121 (84.0)	92 (76.0)	29 (24.0)	0.818
No	—	—	—	—	23 (16.0)	18 (78.3)	5 (21.7)	

p*: Statistical tests: Pearson’s χ² test or Fisher’s exact test, as appropriate. BMI: Body Mass Index

Among participants with diabetes mellitus, dynapenia was more frequent in men (*p* = 0.013), older people (*p* = 0.009), those with multimorbidity (*p* = 0.003), depressive symptoms (*p* = 0.019), cognitive decline (*p* = 0.003), and physical inactivity (*p* = 0.003).

In the univariate analysis, being female and engaging in physical activity reduced the odds of dynapenia in both individuals without diabetes mellitus and those with diabetes mellitus. In contrast, being aged 80 years or older, presenting depressive symptoms, and having cognitive decline increased the odds of dynapenia in both groups. Having four or fewer years of education, being eutrophic or overweight, alcohol consumption, and intake of meat and fruits were associated with dynapenia only among participants without diabetes mellitus. Meanwhile, being aged 70–79 years, having multimorbidity, and higher HbA1c levels were associated with dynapenia only among participants with diabetes mellitus. (Table 3).

**Table 3:** Univariable analysis: crude odds ratios (OR) for dynapenia by individual characteristics, stratified by diabetes status

Variable	Without Diabetes OR (95% CI)	With Diabetes OR (95% CI)
Sex		
Male	1.00	1.00
Female	0.51 (0.27–0.93)	0.37 (0.16–0.82)
Age group		
60–69 years	1.00	1.00
70–79 years	1.83 (0.90–3.74)	2.68 (1.06–6.71)
80 years or older	6.18 (2.82–13.53)	5.60 (1.70–18.38)
Years of schooling		
> 4 years	1.00	1.00
≤ 4 years	2.35 (1.19–4.67)	1.21 (0.52–2.82)
Living arrangement		
Does not live alone	1.00	1.00
Lives alone	0.97 (0.48–1.96)	0.27 (0.06–1.26)
Household income		
> 2 minimum wages	1.00	1.00
> 1 to ≤ 2 minimum wages	1.87 (0.90–3.86)	0.76 (0.31–1.83)
≤ 1 minimum wage	2.21 (0.98–4.95)	0.86 (0.28–2.69)
Multimorbidity		
No	1.00	1.00
Yes	0.97 (0.72–1.31)	0.42 (0.23–0.78)
Depressive symptoms		
None	1.00	1.00
Present	2.58 (1.43–4.65)	2.50 (1.14–5.48)
Cognitive decline		
No	1.00	1.00
Yes	3.34 (1.82–6.11)	3.30 (1.48–7.38)
BMI		
Underweight	1.00	1.00
Normal weight	0.24 (0.10–0.59)	1.82 (0.34–9.63)
Overweight	0.27 (0.12–0.61)	1.14 (0.22–5.74)
Physical activity		
No	1.00	1.00
Yes	0.27 (0.12–0.61)	0.17 (0.04–0.60)
Smoking		
No	1.00	1.00
Yes	1.02 (0.43–2.39)	1.52 (0.43–5.30)
Alcohol consumption		
No	1.00	1.00
Yes	0.28 (0.13–0.62)	0.37 (0.12–1.14)
Milk consumption		
Yes	1.00	1.00
No	0.82 (0.43–1.57)	0.94 (0.39–2.23)
Meat consumption		
Yes	1.00	1.00
No	2.99 (1.61–5.54)	0.88 (0.35–2.16)
Fruit consumption		
Yes	1.00	1.00
No	2.57 (1.38–4.80)	1.12 (0.43–2.92)
Legume consumption		
Yes	1.00	1.00
No	1.13 (0.42–3.02)	1.09 (0.21–5.68)
HbA1c	0.71 (0.36–1.39)	1.22 (1.02–1.47)
Diabetes medication use		
No	–	1.00
Yes	–	1.13 (0.38–3.32)

OR = Odds Ratio; 95% CI = 95% Confidence Interval

In the multivariable analysis, being female and engaging in physical activity reduced the likelihood of dynapenia in both individuals with and without diabetes mellitus. Advanced age and depressive symptoms increased the likelihood of dynapenia in both groups. Among individuals with diabetes mellitus, elevated HbA1c levels were associated with a higher probability of dynapenia (*p* = 0.017). In individuals without diabetes mellitus, no significant association was observed between glycemic levels and dynapenia (Table 4).

**Table 4:** Multivariable analysis: adjusted OR for factors associated with dynapenia by diabetes status (final models)

Variable (reference)	Adjusted OR (95% CI) – Without diabetes	*p*	Adjusted OR (95% CI) – With diabetes	*p*
HbA1c (per + 1% point)	0.63 (0.30–1.32)	0.224	1.34 (1.05–1.71)	0.017
Sex (female vs. male)	0.43 (0.21–0.89)	0.023	0.25 (0.09–0.70)	0.008
Age 70–79 vs. 60–69	2.35 (1.06–5.18)	0.034	2.74 (0.92–8.12)	0.069
Age ≥ 80 vs. 60–69	5.52 (2.36–12.90)	< 0.001	8.52 (1.86–39.10)	0.006
Depressive symptoms	2.98 (1.51–5.86)	0.002	4.03 (1.47–11.08)	0.007
Physical activity (yes)	0.30 (0.12–0.71)	0.007	0.18 (0.04–0.74)	0.018

*OR: Adjusted Odds Ratio from multivariate logistic regression analysis.p: Statistical significance value from the multivariate model. 95% +CI: 95% Confidence Interval for the Odds Ratio

**Fig. 1:**
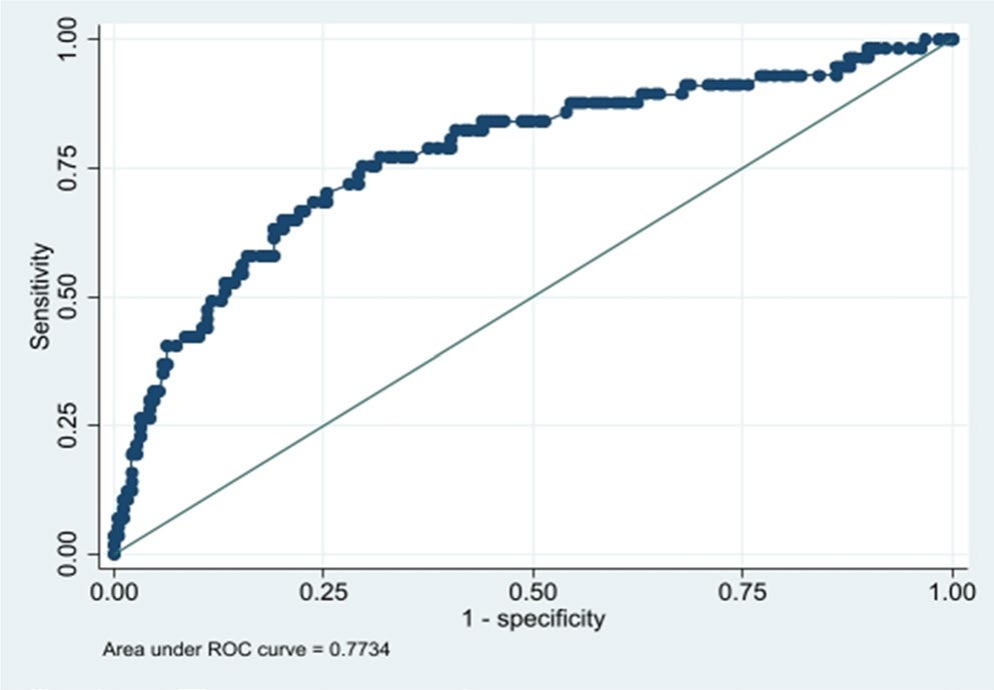
ROC curve for the multivariate model of dynapenia in older people without diabetes

Finally, Fig. 1 presents the ROC curve of the regression model for the group without diabetes mellitus, with an area under the curve (AUC) of 0.77, indicating good discriminatory power.

**Fig. 2:**
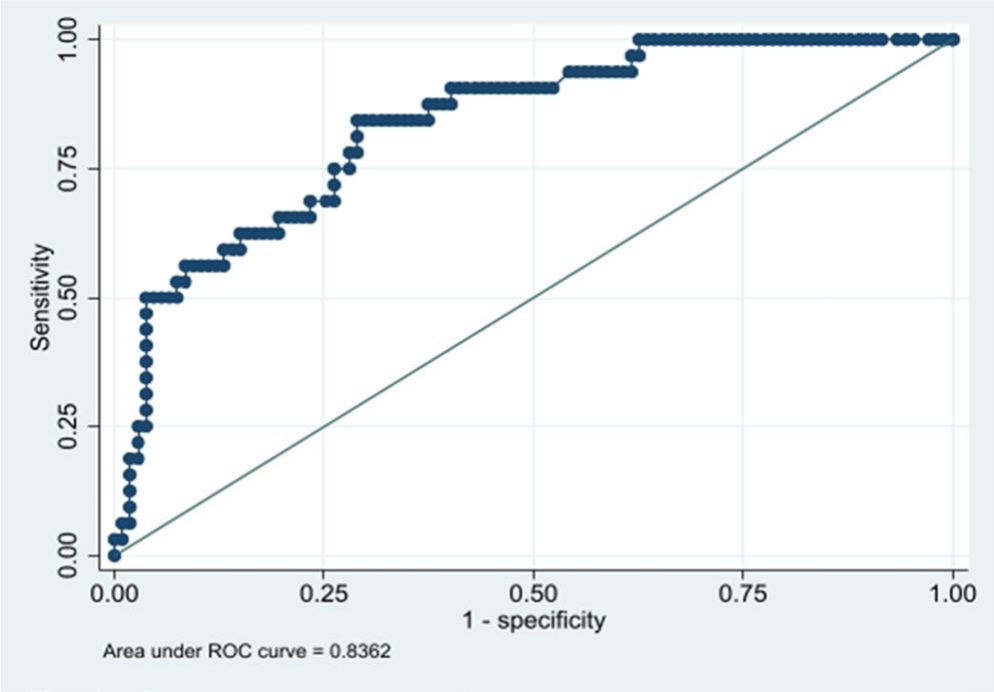
ROC curve for the multivariate model of dynapenia in older people with diabetes

For the group with diabetes mellitus (Fig. 2), the AUC was 0.83, suggesting excellent discriminative ability of the model for the presence of dynapenia.

## Discussion

The aim of this study was to investigate the association between HbA1c levels and dynapenia in community-dwelling older people with and without diabetes mellitus. The main findings indicate that elevated HbA1c levels are significantly associated with dynapenia only in older people with diabetes mellitus, with dynapenic individuals presenting higher median HbA1c values [7.4% (IQR: 6.5–9.0)] compared with their non-dynapenic counterparts [6.9% (IQR: 6.1–8.0)]. This result suggests that chronic hyperglycemia may accelerate the decline in muscle strength, contributing to reduced functionality in this population, even after adjustment for socioeconomic, health, and lifestyle variables.

Several previous studies support the relationship between chronic hyperglycemia and muscle loss in older people with diabetes mellitus. Sustained hyperglycemia has been associated with significant reductions in muscle strength in different diabetic older populations, indicating the consistency of this association across various settings [13, 27, 28]. Moreover, evidence shows that elevated HbA1c levels are directly related to reduced handgrip strength in older people, corroborating our findings [29].

Pathophysiological mechanisms involving advanced glycation end products (AGEs) and chronic inflammatory processes may partly explain the observed association between elevated HbA1c and dynapenia in diabetic older individuals [30, 31]. These mechanisms promote neuromuscular and structural damage, directly contributing to accelerated muscle and functional decline in this specific group [32].

Conversely, older people without diabetes mellitus showed no association between moderately elevated HbA1c levels and dynapenia. This result aligns with studies indicating that the mild physiological increase in HbA1c due to healthy aging does not reach a pathological threshold capable of inducing significant muscle changes, and that mild metabolic alterations, such as age-related insulin resistance and reduced erythrocyte turnover, do not appear to have a sufficient impact on muscle strength in older populations without diabetes mellitus [33, 34]. Additionally, the deleterious effects of glucose on muscle and nerves are clinically relevant only under conditions of chronic and elevated exposure, typical of uncontrolled diabetes mellitus [35, 36]. Regarding sex differences, the results indicate that female sex was associated with a lower risk of dynapenia, which may be related to differences in muscle composition and age-related trajectories of muscle decline between men and women. Evidence suggests that muscle power declines linearly with aging and that this reduction is more pronounced among men [37]. Similarly, studies assessing handgrip strength have shown consistent age-related decline patterns in men [38].

Beyond age-related changes, metabolic and biological factors may further contribute to these sex differences. Uncontrolled diabetes mellitus has been associated with greater muscle strength loss, with a more pronounced impact among men [39]. Differences in muscle fiber composition may partially explain this finding, as women tend to present a higher proportion of type I muscle fibers, which are more resistant to atrophy [40]. Furthermore, women appear to be less susceptible to muscle loss induced by inflammation when compared with men [41].

However, biological mechanisms alone may not fully account for the observed association in our study. Although most studies report a higher prevalence of dynapenia among older women, in our community-based sample, female sex was associated with a lower likelihood of dynapenia. This finding may reflect the specific profile of the study population, in which older women are often more engaged in health services, social programs, and daily functional activities, potentially resulting in greater cumulative physical activity and better preservation of muscle strength.

In line with this interpretation, behavioral factors also emerged as relevant in our analysis. Among the protective factors identified, regular physical activity stands out. In the present study, physical activity was inversely associated with dynapenia in both older people with and without diabetes mellitus, reinforcing its potential role in preserving muscle function. Previous evidence indicates that resistance exercise improves muscle strength, contributes to HbA1c reduction, and enhances muscle quality and neuromuscular performance in older people with diabetes mellitus [42]. In particular, resistance training enhances motor unit recruitment and neuromuscular efficiency, which may help mitigate age-related muscle strength decline, regardless of glycemic status [36]. Advanced age was also strongly associated with dynapenia. Previous studies have emphasized that older people in advanced age groups are at increased risk of this condition, likely due to greater biological and functional vulnerability associated with aging [43, 44, 45].

The negative influence of mental health on muscle strength, represented by the association between depressive symptoms and dynapenia in this study, is also supported by the literature. Previous studies have indicated a bidirectional relationship between depression and reduced muscle strength, with individuals presenting persistent depressive symptoms being at higher risk of sarcopenia and dynapenia over time [46, 47, 48].

Despite the consistent results found, this study has limitations that should be considered. Although the sample was probabilistic and representative of community-dwelling older people in an urban Brazilian municipality, the findings should not be generalized to hospitalized or institutionalized older populations, which may present different clinical and functional profiles. The cross-sectional design limits causal inference, hindering the temporal understanding between exposure and outcome. Additionally, the use of self-reported interviews for depressive symptoms and physical activity may introduce information bias. In addition, dietary intake was assessed using the frequency of food group consumption, without quantification of energy or specific nutrient intake, which limits the nutritional interpretation of the findings. The exclusion of older people with severe cognitive or physical impairments may also have limited the generalizability of the findings.

Among the strengths of this study is the direct comparative analysis between older people with and without diabetes mellitus and the methodological rigor in the assessment of HbA1c levels and muscle strength. The results offer important contributions to clinical practice and public health policies aimed at preserving functionality in diabetic older people.

These findings reinforce the importance of multiprofessional attention to glycemic control in older people with diabetes mellitus, aiming to prevent functional loss. Multidimensional approaches, including the promotion of regular physical activity and the maintenance of emotional well-being, are essential for preserving independence and quality of life in this population. New longitudinal studies are recommended to clarify the trajectory and mechanisms involved in the association between HbA1c and muscle loss related to diabetes mellitus in older people.

## Data Availability

The datasets generated and/or analyzed during the current study are available from the corresponding author on reasonable request.

## Declarations

### Clinical trial registration

Clinical trial number: not applicable.
